# Supplementary material for: Engineered Mesenchymal Stem Cells Over-Expressing BDNF Protect the Brain from Traumatic Brain Injury-Induced Neuronal Death, Neurological Deficits, and Cognitive Impairments
Source: Pharmaceuticals (Basel). 2023 Mar 13;16(3):436. doi: 10.3390/ph16030436 (PMC10054459; doi:10.3390/ph16030436)
Supplement: Supplementary file 1 [file pharmaceuticals-16-00436-s001.zip › pharmaceuticals-2219103-supplementary/Supplementary Files for Pharmaceuticals.pdf]

Supplementary information for Pharmaceuticals

Engineered mesenchymal stem cells over-expressing BDNF protect the brain from traumatic brain injury-induced neuronal death, neurological deficits, and cognitive impairments

Bo Young Choi [1,2,†], Dae Ki Hong [3,†], Beom Seok Kang [4], Si Hyun Lee [4], Seunghyuk Choi [4], Hyo-Jin Kim [5], Soon Min Lee [5,*] and Sang Won Suh [4,*]

1 Department of Physical Education, Hallym University, Chuncheon 24252, Republic of Korea
2 Institute of Sports Science, Hallym University, Chuncheon 24252, Republic of Korea
3 Department of Pathology and Laboratory Medicine, Emory University School of Medicine, Atlanta, Georgia, 30322, USA
4 Department of Physiology, Hallym University College of Medicine, Chuncheon 24252, Republic of Korea
5 SL BiGen, Inc., SL BIGEN Research Hall, 85, Songdogwahak-ro, Yeonsu-gu, Incheon 21983, Republic of Korea
* Correspondence: smlee@slbigen.com (S.M.L.); swsuh@hallym.ac.kr (S.W.S.)
† These authors contributed equally to this work.

This file includes:

Figure S1. *In vitro* characterization of BDNF-eMSCs.

Table 1. Proteomic analysis of supernatant of BDNF-eMSCs compared to naïve MSCs.

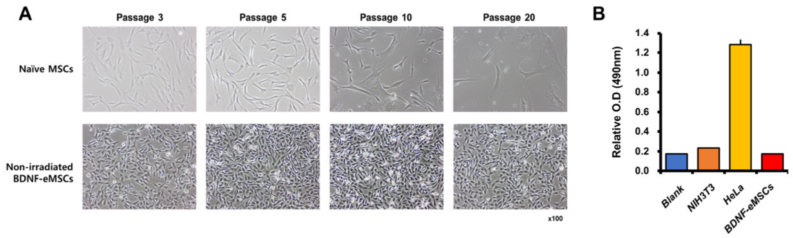

Figure S1. *In vitro* characterization of BDNF-eMSCs. (**A**) Representative image of morphological change of naïve MSCs and BDNF-eMSCs after long-term culture. (**B**) Representative result of *in vitro* tumorigenicity assay of BDNF-eMSCs.

Table S1. Proteomic analysis of supernatant of BDNF‐eMSCs compared to naïve MSCs. Significantly up‐ or down‐regulated BDNF‐eMSC secretome proteins are listed.

UniProtID	Gene_symbol	EntrezID	Protein_description	Up/Down
P23560	BDNF	627	Brain-derived neurotrophic factor	Up
Q99988	GDF15	9518	Growth/differentiation factor 15	Up
P16870	CPE	1363	Carboxypeptidase E	Up
P48745	CCN3	4856	CCN family member 3	Up
P17931	LGALS3	3958	Galectin-3	Up
P53597	SUCLG1	8802	Succinate-CoA ligase [GDP-forming] subunit alpha, mitochondrial	Up
P04066	FUCA1	2517	Tissue alpha-L-fucosidase	Up
P43490	NAMPT	10135	Nicotinamide phosphoribosyltransferase	Up
Q99674	CGREF1	10669	Cell growth regulator with EF hand domain protein 1	Up
Q8IWU5	SULF2	55959	Extracellular sulfatase Sulf-2	Up
P07602	PSAP	5660	Prosaposin	Up
Q9NRA1	PDGFC	56034	Platelet-derived growth factor C	Up
Q9UHL4	DPP7	29952	Dipeptidyl peptidase 2	Up
P30740	SERPINB1	1992	Leukocyte elastase inhibitor	Up
P01033	TIMP1	7076	Metalloproteinase inhibitor 1	Up
Q14517	FAT1	2195	Protocadherin Fat 1	Up
O14960	LECT2	3950	Leukocyte cell-derived chemotaxin-2	Up
P25445	FAS	355	Tumor necrosis factor receptor superfamily member 6	Up
O00748	CES2	8824	Cocaine esterase	Up
P41250	GARS1	2617	Glycine--tRNA ligase	Up
Q9HAT2	SIAE	54414	Sialate O-acetylesterase	Up
Q9UKU9	ANGPTL2	23452	Angiopoietin-related protein 2	Up
P02649	APOE	348	Apolipoprotein E	Up
Q13287	NMI	9111	N-myc-interactor	Up
Q16787	LAMA3	3909	Laminin subunit alpha-3	Up
P13497	BMP1	649	Bone morphogenetic protein 1	Up
Q13219	PAPPA	5069	Pappalysin-1	Up
P07686	HEXB	3074	Beta-hexosaminidase subunit beta	Up
P50454	SERPINH1	871	Serpin H1	Up
Q9H173	SIL1	64374	Nucleotide exchange factor SIL1	Up
P13987	CD59	966	CD59 glycoprotein	Up
O00468	AGRN	375790	Agrin	Up

P20062	TCN2	6948	Transcobalamin-2	Up
O75718	CRTAP	10491	Cartilage-associated protein	Up
B9A064	IGLL5	100423062	Immunoglobulin lambda-like polypeptide 5	Down
O00622	CCN1	3491	CCN family member 1	Down
O43854	EDIL3	10085	EGF-like repeat and discoidin I-like domain-containing protein 3	Down
O60462	NRP2	8828	Neuropilin-2	Down
O60507	TPST1	8460	Protein-tyrosine sulfotransferase 1	Down
O95450	ADAMTS2	9509	A disintegrin and metalloproteinase with thrombospondin motifs 2	Down
O95965	ITGBL1	9358	Integrin beta-like protein 1	Down
P00740	F9	2158	Coagulation factor IX	Down
P00747	PLG	5340	Plasminogen	Down
P01008	SERPINC1	462	Antithrombin-III	Down
P01011	SERPINA3	12	Alpha-1-antichymotrypsin	Down
P01876	IGHA1	3493	Immunoglobulin heavy constant alpha 1	Down
P02452	COL1A1	1277	Collagen alpha-1(I) chain	Down
P02647	APOA1	335	Apolipoprotein A-I	Down
P02655	APOC2	344	Apolipoprotein C-II	Down
P02768	ALB	213	Serum albumin,Albumin	Down
P02787	TF	7018	Serotransferrin	Down
P03956	MMP1	4312	Interstitial collagenase	Down
P04114	APOB	338	Apolipoprotein B-100	Down
P05155	SERPING1	710	Plasma protease C1 inhibitor	Down
P05452	CLEC3B	7123	Tetranectin	Down
P07585	DCN	1634	Decorin	Down
P07996	THBS1	7057	Thrombospondin-1	Down
P08123	COL1A2	1278	Collagen alpha-2(I) chain	Down
P08572	COL4A2	1284	Collagen alpha-2(IV) chain	Down
P09668	CTSH	1512	Pro-cathepsin H	Down
P09871	C1S	716	Complement C1s subcomponent	Down
P10124	SRGN	5552	Serglycin	Down
P10915	HAPLN1	1404	Hyaluronan and proteoglycan link protein 1	Down
P12110	COL6A2	1292	Collagen alpha-2(VI) chain	Down
P12111	COL6A3	1293	Collagen alpha-3(VI) chain	Down
P13612	ITGA4	3676	Integrin alpha-4	Down
P14543	NID1	4811	Nidogen-1	Down
P17936	IGFBP3	3486	Insulin-like growth factor-binding protein 3	Down
P19801	AOC1	26	Amiloride-sensitive amine oxidase [copper-containing]	Down
P19823	ITIH2	3698	Inter-alpha-trypsin inhibitor heavy chain H2	Down

P21246	PTN	5764	Pleiotrophin	Down
P21810	BGN	633	Biglycan	Down
P22692	IGFBP4	3487	Insulin-like growth factor-binding protein 4	Down
P23142	FBLN1	2192	Fibulin-1	Down
P26583	HMGB2	3148	High mobility group protein B2	Down
P27658	COL8A1	1295	Collagen alpha-1(VIII) chain	Down
P28300	LOX	4015	Protein-lysine 6-oxidase	Down
P29279	CCN2	1490	CCN family member 2	Down
P35442	THBS2	7058	Thrombospondin-2	Down
P35443	THBS4	7060	Thrombospondin-4	Down
P35555	FBN1	2200	Fibrillin-1	Down
P36955	SERPINF1	5176	Pigment epithelium-derived factor	Down
P43652	AFM	173	Afamin	Down
P54289	CACNA2D1	781	Voltage-dependent calcium channel subunit alpha-2/delta-1	Down
P58397	ADAMTS12	81792	A disintegrin and metalloproteinase with thrombospondin motifs 12	Down
Q08431	MFGE8	4240	Lactadherin	Down
Q12805	EFEMP1	2202	EGF-containing fibulin-like extracellular matrix protein 1	Down
Q12841	FSTL1	11167	Follistatin-related protein 1	Down
Q12860	CNTN1	1272	Contactin-1	Down
Q13443	ADAM9	8754	Disintegrin and metalloproteinase domain-containing protein 9	Down
Q13822	ENPP2	5168	Ectonucleotide pyrophosphatase/phosphodiesterase family member 2	Down
Q14314	FGL2	10875	Fibroleukin	Down
Q14767	LTBP2	4053	Latent-transforming growth factor beta-binding protein 2	Down
Q16270	IGFBP7	3490	Insulin-like growth factor-binding protein 7	Down
Q68BL8	OLFML2B	25903	Olfactomedin-like protein 2B	Down
Q6UXH9	PAMR1	25891	Inactive serine protease PAMR1	Down
Q7Z7G0	ABI3BP	25890	Target of Nesh-SH3	Down
